# Vulnerability in older adults in Primary Health Care: challenges associated with frailty and functionality

**DOI:** 10.1590/0034-7167-2024-0219

**Published:** 2025-10-03

**Authors:** Cláudia Martins da Costa, Kellen Rosa Coelho, Patrícia Peres de Oliveira, Renata Cristina Silva, Carmelo Sergio Gómez Martínez, Gilson de Vasconcelos Torres, Clarissa Terenzi Seixas, Thalyta Cristina Mansano Schlosser

**Affiliations:** I Universidade Federal de São João del-Rei. Divinópolis, Minas Gerais, Brazil; II Universidade Estadual de Campinas. Campinas, São Paulo, Brazil; III Universidad Católica San Antonio de Murcia. Murcia, Spain; IV Universidade Federal do Rio Grande do Norte. Natal, Rio Grande do Norte, Brazil; V Université Paris Cité. Paris, France

**Keywords:** Frailty, Health Vulnerability, Functional Status, Aged, Primary Health Care, Fragilidad, Vulnerabilidad en Salud, Estado Funcional, Anciano, Atención Primaria de Salud.

## Abstract

**Objectives:**

to analyze the association between vulnerability and levels of frailty and functionality of older adults assisted by primary care in a medium-sized municipality in the Midwest of Minas Gerais.

**Methods:**

a cross-sectional, analytical, quantitative observational study, carried out in a multicenter study setting of the International Research Network on Vulnerability, Health, Safety and Quality of Life of Older Adults. To assess the associations between levels of frailty and qualitative variables, Pearson’s chi-square test and modified Poisson regression models were applied, with robust variance, considering a significance level of 5%.

**Results:**

150 older adults participated in the study. A strong association was identified between functional and cognitive decline, vulnerability, and an increased likelihood of developing frailty syndrome.

**Conclusions:**

an association was evidenced between the presence of vulnerability in older adults with a higher level of dependence in basic and instrumental activities and the presence or risk of frailty.

## RESUMEN

**Objetivos:**

analizar la asociación entre vulnerabilidad y niveles de fragilidad y funcionalidad de personas mayores atendidas por la atención primaria en un municipio de mediano tamaño del Centro-Oeste de Minas Gerais.

**Métodos:**

estudio observacional cuantitativo, analítico, transversal, realizado en un escenario que forma parte de un estudio multicéntrico de la Red Internacional de Investigación sobre Vulnerabilidad, Salud, Seguridad y Calidad de Vida en las Personas Mayores. Para evaluar asociaciones entre niveles de fragilidad y variables cualitativas se aplicó la prueba chi-cuadrado de Pearson y modelos de regresión de Poisson modificados, con varianza robusta, considerando un nivel de significancia del 5%.

**Resultados:**

participaron del estudio 150 ancianos. Se identificó una fuerte asociación entre el deterioro funcional y cognitivo, la vulnerabilidad y una mayor probabilidad de desarrollar síndrome de fragilidad.

**Conclusiones:**

se evidenció asociación entre la presencia de vulnerabilidad en personas mayores con mayor nivel de dependencia en actividades básicas e instrumentales y la presencia o riesgo de fragilidad.

## INTRODUCTION

Brazil is undergoing an intense demographic transition, in which fertility rates are decreasing while the number of older Brazilian adults, especially those who are long-lived, is increasing. The Brazilian population was estimated at 217.7 million people in 2022, with 14.7% of this number being older adults, which represents approximately 31.2 million Brazilians. According to estimates by the Brazilian Institute of Geography and Statistics for the year 2060, Brazil will have approximately 58.2 million older adults, which will account for 25.5% of the Brazilian population^(1)^.

Regarding aging, senescence is a physiological process unrelated to disease. Therefore, pathological aging associated with various changes resulting from chronic diseases can be avoided or delayed by adopting healthy lifestyle habits and behaviors^(2)^, although this process is multicausal and changing habits is no guarantee of physiological aging. The Brazilian National Health Policy for Older Adults aims to promote healthy aging through, among other things, comprehensive healthcare for older adults^(3,4)^.

Among the diseases and conditions associated with unhealthy aging, cognitive and functional declines can trigger a process of weakening and dependence in older adults to perform basic activities of daily living (BADL) and instrumental activities of daily living (IADL) gradually. As a consequence, older adults lose the ability to perform self-care and autonomy, i.e., the ability to make decisions on their own. When there is vulnerability, this decline can be even more accelerated^(5,6)^.

Vulnerability is a polysemic concept that many authors have sought to define in recent decades. In this text, we will adopt an understanding shared by Ayres^(7)^: vulnerability as exposure to risks and diverse needs, resulting from multiple determinations that permeate life contexts of citizens who face frail access to rights. Its character is multidimensional, and its nature is psychosocial, socioeconomic and biological. From this perspective, vulnerability is conceptualized in three interconnected dimensions: individual, social and programmatic. These factors are involved in the process of health and illness/injuries^(7)^.

In the individual dimension, subjects are understood in their particularities and interpersonal interactions. In relation to the social dimension, religious beliefs, cultural aspects, family structure and access to basic services predominate. The programmatic dimension refers to government institutions and healthcare services as well as the way in which they act to reduce inequities that result in vulnerability^(6,7)^.

The aging process is also characterized by changes at the molecular level. These changes, combined with exposure to vulnerable conditions, lead to the incidence of comorbidities, which can result in the process of frailty in older adults. Frailty is a multifactorial clinical syndrome characterized by functional decline, reduced functional reserve of multiple systems, and deterioration of health. The frailty syndrome in older adults is considered a health concern that presupposes undesirable outcomes, such as risk of malnutrition, falls, hospitalization, functional disability, institutionalization, and death^(8,9)^.

Frailty syndrome has become a public health problem as it has significant repercussions on health planning and care by leading to a high demand for medical care, examinations and home care. Technological, human, financial, material and structural resources are not adequate to meet the demands presented in this new scenario^(10)^.

The aging and longevity process is not always accompanied by a healthy life, which leads to a greater risk of vulnerability, illness and frailty in older adults. Primary Health Care (PHC), as the gateway to the health system and its proximity to the population, receives the impacts resulting from older adults’ vulnerability. This, in turn, highlights the need to identify older adults at risk of vulnerability, frailty and functional decline in the community early on^(10)^.

Given the above, this study is justified by the increase in the elderly population and the growing need to investigate factors associated with vulnerability, functional decline and frailty syndrome in older adults, in order to support healthcare professionals, especially in PHC, to develop strategies to promote health and prevent health problems in the elderly population.

## OBJECTIVES

To analyze the association between vulnerability and levels of frailty and functionality of older adults assisted by PHC in a medium-sized municipality in the Midwest of Minas Gerais, Brazil.

## METHODS

### Ethical aspects

It is important to emphasize that the study was conducted in accordance with the guidelines and regulatory standards for research involving human beings set forth in the Brazilian National Health Council, Resolution 466, of December 12, 2012. The multicenter project was submitted to and approved by the respective Research Ethics Committees of *Hospital Universitário Onofre Lopes* (*Universidade Federal do Rio Grande do Norte*) *and Universidade Federal de São João del-Rei*. Study participants were informed about the objectives, procedures, and importance of the study. The Informed Consent Form was obtained from all individuals involved in the study through a written document. Participants’ anonymity and privacy were guaranteed as well as their right to continue or withdraw at any time during data collection.

### Study design

This is an observational, cross-sectional, analytical, quantitative study to investigate the vulnerability and frailty in health of older adults assisted by PHC. To guide this study, the guideline STrengthening the Reporting of OBservational studies in Epidemiology was used^(11)^.

### Study location

Data collection was performed in the PHC setting of a municipality located in the Midwest of Minas Gerais, Brazil. The participating municipality is medium-sized and has 242,328 inhabitants, whose population has a monthly income of 2.1 minimum wages. The municipality has 51 Family Health Strategy (FHS) teams, accounting for 71.06% of family health coverage. This setting is part of a multicenter study in the International Research Network on Vulnerability, Health, Safety and Quality of Life of Older Adults in Brazil, Portugal, Spain and France.

### Period

Data collection was carried out from July to December 2022.

### Population and sample

The population was composed of older adults aged 60 or over, in accordance with the Statute of the Elderly^(5)^, with preserved cognitive capacity, registered and users of the municipality’s PHC care units.

To calculate the sample size of participants, considering an estimated target population of 2,083 older adults using PHC, a 95% confidence level (Z=1.96), sampling error (e=0.08), estimated proportion of expected hits (P) of 50% and expected error (Q) of 50% of older adults served in PHC, the estimated sample was 150 older adults.

The inclusion criteria were being 60 years of age or older, being registered in a PHC health unit and not having cognitive impairment assessed by the Mini Mental State Examination (MMSE) questionnaire^(12)^. As this assessment is influenced by the educational level factor, for this study, the following values were used as the cut-off point for educational levels: 18 points (illiterate), 21 (elementary education) and 24 (high school or higher education)^(13)^. The exclusion criteria adopted were having a medical diagnosis of intellectual, neurological or mental disability that could hinder cognitive and motor tests.

### Study design

The participating older adults were pre-selected by convenience in the PHC units that had the largest number of older adults registered in the FHS of the municipality under study, which totaled 14 units. Given the list of older adults indicated by the respective health teams of predefined locations, participant recruitment was carried out in the units themselves, when they sought healthcare and/or during home visits.

Data collection was performed by a master’s student nurse and four nursing students, all previously trained. Data collection was conducted through interviews conducted in a private environment, both in PHC units and in the patients’ own homes. Each interview lasted approximately 90 minutes, totaling 13,500 minutes at the end of data collection. Data collection instruments were organized in Google Forms^®^ to optimize data storage and subsequent organization.

The instruments used in the interviews were a sociodemographic questionnaire (sex, reported race, marital status, education, family income and occupation), prepared by the authors, and MMSE, which consists of several items that assess cognitive functions, used as a cognitive screening tool, such as orientation to time and place (5 points), immediate recall (3 points), attention and calculation (5 points), recall (5 points), word recall (3 points), language (8 points) and construct ability (1 point), ranging from a minimum (0) to a maximum (30). The scoring was performed as follows: 30 to 26 points (preserved cognitive functions); 26 to 24 points (change not suggestive of deficit); and 23 points or less (suggestive of cognitive deficit). For this study, only older adults with a score ≥ 17 points were included^(12,13)^.

Frailty was assessed using the Edmonton Frailty Scale, which assesses nine aspects, such as cognition, general health status, functional independence, social support, medication use, nutrition, mood, continence and functional performance, investigated by 11 items. Regarding the score, 17 represents the highest level of frailty. The scores for frailty analysis are: 0-4, not frailty; 5-6, vulnerable; 7-8, mild frailty; 9-10, moderate frailty; 11 or more, severe frailty^(14)^.

The Self-Reported Frailty Scale considers the self-perception of older adults or caregivers/informants regarding the components that characterize the frailty syndrome. The perception of unintentional weight loss, reduced strength, presence of fatigue, low physical activity and reduced walking speed is assessed through the following classification: presence of three or more criteria (presence of frailty); one or two criteria (pre-frailty); and no criteria (not frail)^(15)^.

To assess functional vulnerability, the Vulnerable Elders Survey (VES-13) was used, which is included in the older adult handbook. It is a simple instrument designed to identify older adults at risk of frailty and death. The VES-13 consists of 13 items, which cover age, self-reported health, functional capacity and autonomy in performing routine activities, and its score ranges from 0 to 13 points, with a score equal to or greater than three considered the cut-off point to classify individuals as vulnerable^(16)^.

The Barthel Functioning Questionnaire assesses functional independence in self-care across ten basic tasks. Each item is scored based on patients’ performance in performing tasks independently, with some assistance, or dependently. An overall score is formed by assigning points in each category, depending on the time and assistance required by each patient. The score ranges from 0 to 100, in five-point intervals, with higher scores indicating greater independence^(17)^.

Concerning functionality assessment, the Lawton and Brody Functionality Questionnaire was also applied, which aims to assess older adults’ autonomy in carrying out activities necessary for living independently in the community. The ability to use the telephone, shop, prepare meals and housework, travel, manage finances and use medications independently is assessed. Each item assessed varies between 1 and 3. A score of 3 indicates that the interviewee is in a condition of independence; a score of 2 expresses a condition of semi-dependence, which indicates that the participant needs partial help with IADLs; and a score of 1 demonstrates the existence of total dependence. To finalize assessment, the scores of all items are added together: the maximum score is 21, which indicates total independence on the part of the individual. The minimum score is 7, which, in turn, demonstrates that the person being assessed is completely dependent^(18,19)^.

The Risk of Functional Decline Questionnaire, PRISMA 7, consists of seven self-reported items to identify frailty. The items are composed of domains such as sex, age, independence and functionality. The answer to the questions is dichotomous: “yes” (1 point) or “no” (0 points). The sum of the answers varies between 0 and 7, and a score ≥3 may indicate the presence of frailty^(20)^.

### Analysis of results, and statistics

The data were organized in an Excel spreadsheet and analyzed using the Statistical Package for the Social Sciences version 23.0 and SAS version 9.4, with the assistance of a statistician. To assess associations between levels of frailty and qualitative variables, Pearson’s chi-square test was applied. For analyses where a significant result was observed in the chi-square test and the qualitative variable presented more than two response categories, modified Poisson regression models with robust variance^(21)^ were applied in order to identify between which response categories there was a statistical difference. The results of these models presented the estimates obtained for Prevalence Ratio (probability of vulnerability), as well as their respective 95% Confidence Intervals, considering a significance level of 5%.

To test the difference between the means of quantitative variables, Student’s t-test for independent and paired samples, ANOVA and Pearson’s correlation tests were used, and linear regression was used in the case of variables with normality, and similar tests, in the case of non-parametric statistics. Logistic regression was performed to determine whether characteristics such as functional capacity, cognitive status and presence of frailty are related to vulnerability. For comparisons between the levels of vulnerability (VES-13) in relation to quantitative variables, the Mann-Whitney test was applied^(22)^. Data distribution was assessed using the Shapiro-Wilk test^(23)^.

## RESULTS

A total of 150 older adults participated in the study. The majority (62.67%) were female; 37.33% were male; and 44.67% declared themselves to be white. The mean age was 70.33 (±7.51) years, and 79.34% were literate. In relation to marital status, 60.66% of participants had a partner and were retired (77.33%). Family income was 2 minimum wages for 43.33% of participants (Table 1).

**Table 1:** Characterization of participants in relation to sociodemographic conditions, 2022 (N 150)

Variable	n	%
Sex		
Female	94	62.67
Male	56	37.33
Reported color		
White	67	44.67
Brown	38	25.33
Black	45	30.00
Marital status		
With partner	91	60.66
Without partner	59	39.33
Level of education		
Illiterate	31	20.67
Literate	119	79.34
Family income		
1 minimum wage (1,212.00)	36	24.00
2 minimum wages (2,424.00)	65	43.33
≥3 minimum wages (3,636.00)	49	32.67
Occupation		
Retired	116	77.33
Houseperson	14	9.33
Unemployed	4	2.67
Formal job	3	2.00
Informal job	13	8.67

Mental status assessment showed that participants did not have cognitive changes suggestive of deficit. Regarding vulnerability assessed by the VES-13, approximately 20.67% of participants were at risk of frailty and 38.67% were already in a state of frailty. The application of the Edmonton Scale showed that the majority of older adults had some degree of frailty and 10% of participants were in severe frailty. According to the self-reported frailty scale, only 4.67% did not present any degree of frailty (Table 2).

**Table 2:** Characterization of participants regarding cognitive preservation, presence of frailty and functional capacity, 2022 (N=150)

Variable	n	%
Mini Mental State Examination		
Illiterate (17 to 20 points)	32	21.33
Literate (20 to 30 points)	118	78
Vulnerable Elders Survey-13		
Not vulnerable	61	40.67
Risk of frailty	31	20.67
Frail	58	38.67
Edmonton Frailty Scale		
Not frail	65	43.33
Vulnerable	33	22.00
Mildly frail	25	16.67
Moderately frail	12	8.00
Severely frail	15	10.00
Self-reported frailty scale		
Non-frail	7	4.67
Pre-frailty	57	38.00
Frailty	86	57.33
Barthel		
Dependence on 50% help with tasks	3	2.00
Dependence on 25% help with tasks	13	8.67
Independent	134	89.33
PRISMA 7		
Not frail	96	64.00
Frail	54	36.00
Lawton and Brody		
Totally dependent	41	27.33
Slightly dependent	48	32.00
Independent	61	40.67

To assess functional capacity in the execution of BADLs, the Barthel Scale was used. It was observed that the majority of participants were considered independent (89.33%), followed by 8.67% of older adults with modified dependence, needing help in 25% of tasks, and 2.00% were considered to have modified dependence and need help in up to 50% of activities (Table 2).

The Lawton and Brody Scale is used to assess IADL, and in this domain, 27.7% were classified as totally dependent. The same proportion of older adults (27.7%) was classified as slightly dependent, and 44.5% of older adults were considered independent (Table 2).

In the assessment of functional capacity for performing IADL and BADL, the association between functional decline and risk of frailty or a state of frailty already established was evident. The same was observed regarding scales for assessing functional decline, self-perception of functional status and assessment of cognitive status (Table 3).

**Table 3:** Association of frailty conditions and functional capacity, 2022 (N=150)

Variable	Vulnerable Elders Survey-13				*p* value^*^	Prevalence Ratio^**^	95% CI	
	Not vulnerable		Vulnerable					
	n	%	n	%			LL	UL
Barthel					0.0030			
Modified dependency	1	6.25	15	93.75				
Independent	60	44.78	74	55.22				
Edmonton Frailty Scale					< 0.0001			
Not frail/vulnerable/slight frailty	61	49.59	62	50.41				
Moderate frailty/severe frailty	0	0.00	27	100.00				
Lawton and Brody					< 0.0001			
Totally dependent	9	21.95	32	78.05		**2.65**	**1.72**	**4.07**
Mild dependence	9	18.75	39	81.25		**2.75**	**1.76**	**4.32**
Independent	43	70.49	18	29.51		1.00 (ref)		
PRISMA 7					< 0.0001			
Not frail	60	62.50	36	37.50				
Frail	1	1.85	53	98.15				
Self-reported frailty scale					< 0.0001			
Non-frail/pre-frail	46	71.88	18	28.13				
Frailty	15	17.44	71	82.56				
Mini Mental State Examination					0.0011			
Illiterate (17-20 points)	27	30.00	63	70.00				
Literate (20-30 points)	34	56.67	26	43.33				

* p value obtained using Pearson’s chi-square test; CI - Confidence Interval; LL - lower limit; LS - upper limit;

** Results obtained using a modified Poisson regression model. The probability of presenting the result “risk of frailty/frail” was estimated.

A strong association was observed between dependence in BADL and IADL and the risk of frailty or a state of frailty. The study showed that older adults identified as mildly dependent have a 2.75 times greater probability of being at risk of frailty, while fully dependent older adults have a 2.65 times greater probability of being frail (Table 3).

## DISCUSSION

This study analyzed the association between vulnerability and levels of frailty and functionality of older adults assisted by PHC. Through this study, it was possible to infer that there is a strong association between functional and cognitive decline and risk of vulnerability and frailty. In addition to this, the presence of dependence of these older adults for BADL and IADL and risk of frailty of these older adults were observed.

The severe frailty identified in this study affects 10% of the participating population, and 38.67% were in a state of vulnerability. The presence of frailty is associated with a high risk of health-related problems, since frailty is causally related to multiple chronic conditions and their diverse consequences, such as decreased balance and gait, muscle weakness and fatigue, weight loss, functional and cognitive decline, leaving older adults person in a state of high vulnerability and at risk of developing frailty^(24,25)^.

Frailty syndrome primarily affects individuals’ health, functional and cognitive capacity, which makes it difficult to perform household management activities, i.e., IADLs. IADLs require greater physical and cognitive preservation for their execution; therefore, their decline precedes the decline in functionality for performing self-care activities, in other words, BADLs. As a result of the progression of frailty syndrome and functional decline, associated with other comorbidities, older adults gradually become dependent, lose autonomy and may become more susceptible to disease aggravation, situations of risk for vulnerability and frailty^(26,27)^.

Previous studies have shown that older adults with reduced functional capacity are more dependent on IADLs and at risk of frailty. A higher prevalence of dependence in household management activities can be observed. In this study, 27.33% of older adults are dependent on IADLs. Dependence in these activities can be a determining factor in the progression of functional and cognitive decline. Consequently, older adults with disabilities in IADLs are subject to numerous factors that expose them to risks of frailty and health problems such as frailty syndrome^(27,28)^.

The study also showed that 10.67% of participants are dependent on self-care activities. Functional decline is a determining factor for dependence, need for care and development of frailty syndrome. Previous studies corroborate and show that functional decline is associated with frailty syndrome. According to a study carried out in the city of Ribeirão Preto, SP, it was possible to observe that, of the 148 older adults who participated in the study, 81.9% presented frailty and partial dependence in IADLs and, consequently, in BADLs^(28)^.

In a previous study conducted by Neri^(29)^ in the city of Campinas, SP, results were found that corroborate the findings of this study, which evidenced the strong association between cognitive decline and risk of frailty or already established frailty. Older adults are more likely to be exposed to vulnerability factors and to suffer from chronic diseases and conditions such as frailty syndrome, which in turn can have undesirable outcomes, such as illness, functional and cognitive decline, institutionalization, hospitalization, and death^(29,30)^.

Older adults’ vulnerability is multifactorial and, therefore, monitoring of conditions that provide a greater risk of developing frailty is recommended^(31)^. Knowing the conditions associated with vulnerability and frailty in older adults in the community can provide a better understanding of the actions and strategies needed to prevent cognitive and functional decline^(32)^. In addition, this study can contribute to future longitudinal studies to assess the comparisons made in the present study and interventions to improve frailty and vulnerability in the older population.

Given the above, it is essential that there be adequate government investment and management to meet the needs of this growing population, seeking to adapt and guarantee access to healthcare services and actions to promote health and prevent diseases. It is urgent to simultaneously provide improvements in professionals’ qualifications, especially regarding surveillance and risk stratification of older adults to identify factors that may trigger frailty syndrome. Enabling actions that reduce vulnerability and allow healthy aging of this growing portion of the Brazilian population requires decision-making in the direction of guaranteeing access and combating health inequities.

### Study limitations

However, it is appropriate to consider some limitations of this study, among which it is worth highlighting that, despite being a multicenter study, the results presented here are from only one of the participating municipalities, since data collection continues to be carried out in other Brazilian municipalities as well as in other countries. It is also worth highlighting that participants were selected by convenience among those who attended PHC units, with a bias towards female older adults, since they are the most frequent users of these spaces. Finally, information provided by the older adults themselves may represent biases, especially those related to self-perception, since these are subjective issues and difficult to verify.

### Contributions to health, nursing, or public policy

Knowledge about the risk situation for vulnerability, frailty and functional decline of older adults in this municipality contributes to knowledge of aspects associated with older adults’ vulnerability and frailty. Through this knowledge, it is possible to develop actions to promote active and healthy aging, and contribute to PHC healthcare professionals’ knowledge.

## CONCLUSIONS

This study made it possible to analyze the relationship and association of vulnerability and frailty conditions with the functional decline of older adults assisted by PHC in a city in the Midwest of Minas Gerais. An association was evidenced between the presence of vulnerability in older adults with a higher level of dependence in BADL and IADL and the presence or risk of frailty.

It is important to invest in healthcare professionals’ qualification so that they are able to identify in advance the social and health conditions that result in functional and cognitive decline and, ultimately, in frailty syndrome, and so that they can outline strategies to prevent frailty in older adults.

## Data Availability

The research data are available within the article.
