# Supplementary material for: Vulnerability in older adults in Primary Health Care: challenges associated with frailty and functionality
Source: Rev Bras Enferm. 2025 Oct 3;78(Suppl 2):e20240219. doi: 10.1590/0034-7167-2024-0219 (PMC12500070; doi:10.1590/0034-7167-2024-0219)
Supplement: Translation

# Translation: Vulnerabilidade em idosos na Atenção Primária à Saúde: desafios associados à fragilidade e funcionalidade

**DOI:** 10.1590/0034-7167-2024-0219pt

**Authors:** Cláudia Martins da Costa, Kellen Rosa Coelho, Patrícia Peres de Oliveira, Renata Cristina Silva, Carmelo Sergio Gómez Martínez, Gilson de Vasconcelos Torres, Clarissa Terenzi Seixas, Thalyta Cristina Mansano Schlosser

**Affiliations:** I Universidade Federal de São João del-Rei. Divinópolis, Minas Gerais, Brasil; II Universidade Estadual de Campinas. Campinas, São Paulo, Brasil; III Universidad Católica San Antonio de Murcia. Murcia, Espanha; IV Universidade Federal do Rio Grande do Norte. Natal, Rio Grande do Norte, Brasil; V Université Paris Cité. Paris, França

**Keywords:** Fragilidade, Vulnerabilidade em Saúde, Estado Funcional, Pessoa Idosa, Atenção Primária à, Saúde

## Abstract

**Objetivos:**

analisar a associação entre vulnerabilidade e níveis de fragilidade e funcionalidade de idosos assistidos pela atenção primária de um município de médio porte do Centro-Oeste de Minas Gerais.

**Métodos:**

estudo observacional transversal, analítico, quantitativo, realizado em cenário integrante de estudo multicêntrico da Rede Internacional de Pesquisa sobre Vulnerabilidade, Saúde, Segurança e Qualidade de Vida do Idoso. Para avaliar as associações entre níveis de fragilidade e variáveis qualitativas, foram aplicados o teste qui-quadrado de Pearson e modelos de regressão de Poisson modificados, com variância robusta, considerado um nível de significância de 5%.

**Resultados:**

participaram do estudo 150 idosos. Foi identificada forte associação entre declínio funcional e cognitivo, vulnerabilidade, e aumento da probabilidade de desenvolvimento da síndrome de fragilidade.

**Conclusões:**

foi evidenciada associação entre a presença de vulnerabilidade em idosos com maior nível de dependência nas atividades básicas e instrumentais e a presença ou risco de fragilidade.

## INTRODUÇÃO

O Brasil passa por uma intensa transição demográfica, em que as taxas de fecundidade diminuem enquanto o número de brasileiros idosos, sobretudo longevos, aumenta. A população brasileira foi estimada em 217,7 milhões de pessoas no ano de 2022, sendo 14,7% desse quantitativo constituído por pessoas idosas, o que representa cerca de 31,2 milhões de brasileiros. Segundo estimativas do Instituto Brasileiro de Geografia e Estatística para o ano de 2060, o Brasil terá aproximadamente 58,2 milhões de idosos, o que representará 25,5% da população brasileira^(1)^.

No que concerne ao envelhecimento, senescência é um processo fisiológico não relacionado ao acometimento por doenças. Destarte, envelhecimento patológico associado a diversas alterações decorrentes de doenças crônicas pode ser evitado ou adiado mediante adoção de hábitos de vida e comportamentos saudáveis^(2)^, ainda que esse processo seja multicausal e a mudança de hábitos não seja garantia de envelhecimento fisiológico. A Política Nacional de Saúde do Idoso visa promover envelhecimento saudável por meio, entre outros, da integralidade da atenção à saúde do idoso^(3,4)^.

Entre as doenças e agravos associados ao envelhecimento não saudável, os declínios cognitivo e funcional podem desencadear um processo de fragilização e dependência do idoso para execução das atividades básicas de vida diária (ABVD) e das atividades instrumentais de vida diária (AIVD) de forma gradativa. Como consequência, o idoso perde a capacidade de realizar o autocuidado e sua autonomia, isto é, a capacidade de tomar decisões por conta própria. Quando existe presença de vulnerabilidade, esse declínio pode ser ainda mais acelerado^(5,6)^.

Vulnerabilidade é um conceito polissêmico que muitos autores buscaram delimitar nas últimas décadas. Neste texto, adotaremos uma compreensão compartilhada por Ayres^(7)^: vulnerabilidade como exposição a riscos e necessidades diversas, fruto de múltiplas determinações que atravessam os contextos de vida de cidadãos que enfrentam frágil acesso a direitos. Seu caráter é multidimensional, e sua natureza, psicossocial, socioeconômica e biológica. Nessa perspectiva, a vulnerabilidade é conceituada em três dimensões interligadas: individual, social e programática. Esses fatores estão envolvidos no processo de saúde e doença/agravos^(7)^.

Na dimensão individual, o sujeito é compreendido em suas particularidades e interações interpessoais. Em relação à dimensão social, predominam as crenças religiosas, aspectos culturais, estrutura familiar e acesso aos serviços básicos. A dimensão programática se refere às instituições governamentais e serviços de saúde, bem como a forma como esses atuam para diminuir as iniquidades que resultam em vulnerabilidade^(6,7)^.

O processo de envelhecimento também é caracterizado por alterações a nível molecular. Essas alterações, aliadas à exposição a condições de vulnerabilidade, propiciam incidência de comorbidades, que podem resultar no processo de fragilização da pessoa idosa. Fragilidade é uma síndrome clínica multifatorial caracterizada pelo declínio funcional, redução da reserva funcional de múltiplos sistemas e deterioração da saúde. A síndrome de fragilidade do idoso é considerada um agravo de saúde que pressupõe desfechos indesejáveis, como risco de desnutrição, queda, hospitalização, incapacidade funcional, institucionalização e morte^(8,9)^.

A síndrome da fragilidade tem se tornado um problema de saúde pública na medida em que tem repercussões significativas no planejamento e na assistência à saúde ao acarretar alta procura por assistência médica, exames e assistência domiciliar. Os recursos tecnológicos, humanos, financeiros, materiais e de estrutura não estão adequados para atender às demandas apresentadas nesse novo cenário^(9,10)^.

O processo de envelhecimento e longevidade nem sempre vem acompanhado de uma vida saudável, o que acarreta maior risco de vulnerabilidade, adoecimento e fragilização da pessoa idosa. A Atenção Primária à Saúde (APS), como porta de entrada para o sistema de saúde e sua proximidade com a população, recebe os impactos resultantes da vulnerabilidade da pessoa idosa. Isso, por sua vez, evidencia a necessidade de se identificar de forma precoce idosos em risco de vulnerabilidade, fragilização e declínio funcional na comunidade^(10)^.

Diante do exposto, este estudo se justifica pelo aumento da população idosa e necessidade crescente de se investigar os fatores associados à vulnerabilidade, declínio funcional e síndrome de fragilidade do idoso, de modo a subsidiar profissionais de saúde, sobretudo na APS, a desenvolver estratégias de promoção da saúde e prevenção de agravos à saúde da população idosa.

## OBJETIVOS

Analisar a associação entre vulnerabilidade e níveis de fragilidade e funcionalidade de idosos assistidos pela APS de um município de médio porte do Centro-Oeste de Minas Gerais, Brasil.

## MÉTODOS

### Aspectos éticos

Ressalta-se que o estudo foi conduzido de acordo com as diretrizes e normas regulamentadoras de pesquisa envolvendo seres humanos que constam no Conselho Nacional de Saúde, Resolução nº 466, de 12 de dezembro de 2012. O projeto multicêntrico foi submetido e aprovado pelos respectivos Comitês de Ética em Pesquisa do Hospital Universitário Onofre Lopes (Universidade Federal do Rio Grande do Norte) e da Universidade Federal de São João del-Rei. Os participantes do estudo foram informados sobre os objetivos, procedimentos e importância do estudo. O Termo de Consentimento Livre e Esclarecido foi obtido de todos os indivíduos envolvidos no estudo por meio de documento escrito. Foram garantidos o anonimato e privacidade do participante, bem como o direito em prosseguir ou desistir em qualquer momento da realização da coleta de dados.

### Tipo de estudo

Trata-se de estudo observacional, transversal, analítico, quantitativo, para investigar a vulnerabilidade e fragilidade em saúde de pessoas idosas assistidas pela APS. Para nortear este estudo, utilizou-se a diretriz *STrengthening the Reporting of OBservational studies in Epidemiology* ^(11)^.

### Local do estudo

A coleta de dados foi realizada no cenário da APS de um município localizado no Centro-Oeste de Minas Gerais, Brasil. O município participante é de médio porte, e tem 242.328 habitantes, cuja população tem uma renda mensal de 2,1 salários mínimos. O município possui 51 equipes de Estratégia Saúde da Família (ESF), representando 71,06% de cobertura de saúde da família. Este cenário faz parte de estudo multicêntrico na Rede Internacional de Pesquisa sobre Vulnerabilidade, Saúde, Segurança e Qualidade de Vida do Idoso no Brasil, Portugal, Espanha e França.

### Período

A coleta de dados foi realizada no período de julho a dezembro de 2022.

### População e amostra

A população foi composta por pessoas idosas na faixa etária igual ou superior a 60 anos, de acordo com o Estatuto do Idoso^(5)^, com capacidade cognitiva preservada, cadastradas e usuárias de unidades de atendimento da APS do município.

Para o cálculo do tamanho amostral dos participantes, considerando uma estimativa da população-alvo de 2.083 idosos usuários da APS, nível de confiança de 95% (Z=1,96), erro amostral (e= 0,08), proporção estimada de acerto esperado (P) de 50% e erro esperado (Q) de 50% de idosos atendidos na APS, a amostra estimada foi de 150 idosos.

Os critérios de inclusão foram ter idade de 60 anos ou mais, ser cadastrado em uma unidade de saúde da APS e não apresentar comprometimento cognitivo avaliado pelo questionário Mini Exame do Estado Mental (MEEM)^(12)^. Como esta avaliação sofre interferência do fator escolaridade, para este estudo, foi utilizado como ponto de corte para os níveis educacionais os seguintes valores: 18 pontos (analfabeto), 21 (educação elementar) e 24 (educação média ou superior)^(13)^. Os critérios de exclusão adotados foram ter um diagnóstico médico de deficiência intelectual, neurológica ou mental que pudesse dificultar os testes cognitivos e motores.

### Desenho do estudo

Os idosos participantes foram pré-selecionados por conveniência nas unidades de APS que tinham o maior quantitativo de idosos cadastrados na ESF do município do estudo, as quais totalizaram 14 unidades. Diante da lista dos idosos indicados pelas respectivas equipes de saúde dos locais predefinidos, o recrutamento dos participantes foi realizado nas próprias unidades, na ocasião em que buscavam atendimento em saúde e/ou em visitas domiciliares.

A coleta de dados foi realizada por uma enfermeira mestranda e por quatro discentes de enfermagem, todos previamente treinados. A coleta foi conduzida por meio de entrevistas realizadas em ambiente privativo, tanto nas unidades da APS quanto nos próprios domicílios. O tempo empregado em cada entrevista foi de aproximadamente 90 minutos, totalizando 13.500 minutos ao final da coleta de dados. Os instrumentos de coleta foram organizados em formulário do tipo *Google Forms^®^*, para otimizar o armazenamento dos dados e posterior organização dos mesmos.

Os instrumentos utilizados nas entrevistas foram questionário sociodemográfico (sexo, cor relatada, estado civil, escolaridade, renda familiar e ocupação), elaborado pelos autores, e MEEM, que é composto por vários itens que avaliam as funções cognitivas, utilizado como ferramenta de triagem cognitiva, como orientação para o tempo (5 pontos), memória imediata (3 pontos), atenção e cálculo (5 pontos), evocação (5 pontos), lembrança de palavras (3 pontos), linguagem (8 pontos) e capacidade construtiva visual (1 ponto), variando de um mínimo (0) até um máximo (30). A pontuação foi realizada da seguinte forma: 30 a 26 pontos (funções cognitivas preservadas); 26 a 24 pontos (alteração não sugestiva de déficit); e 23 pontos ou menos (sugestivo de déficit cognitivo). Para este estudo, foram incluídos apenas idosos com escore ≥ 17 pontos^(12,13)^.

A fragilidade foi avaliada através da Escala de Fragilidade de Edmonton, que avalia nove aspectos, como cognição, estado geral de saúde, independência funcional, suporte social, uso de medicamentos, nutrição, humor, continência e desempenho funcional, investigados por 11 itens. Quanto à pontuação, 17 representa o nível mais elevado de fragilidade. Os escores para análise da fragilidade são: 0-4, não apresenta fragilidade; 5-6, aparentemente vulnerável; 7-8, fragilidade leve; 9-10, fragilidade moderada; 11 ou mais, fragilidade severa^(14)^.

A Escala Autorreferida de Fragilidade considera a autopercepção dos idosos ou cuidadores/informantes com relação aos componentes que caracterizam a síndrome de fragilidade. Avalia-se a percepção sobre perda de peso não intencional, redução da força, presença de fadiga, baixa atividade física e redução da velocidade da caminhada através da seguinte classificação: presença de três ou mais critérios (presença de fragilidade); um ou dois critérios (pré-fragilidade); e nenhum critério (não frágil)^(15)^.

Para avaliar a vulnerabilidade funcional, utilizou-se o *Vulnerable Elders Survey* (VES-13), que está contido na caderneta do idoso. É um instrumento simples elaborado com o objetivo de identificar os idosos com risco de fragilização e morte. O VES-13 é composto por 13 itens, que contemplam idade, saúde autorreferida, capacidade funcional e autonomia na execução de atividades rotineiras, e seu escore varia entre 0 e 13 pontos, sendo a pontuação igual ou maior que três, considerada ponto de corte para classificar o indivíduo como vulnerável^(16)^.

O Questionário de Funcionalidade Escala de Barthel avalia a independência funcional no autocuidado em dez tarefas básicas. Cada item é pontuado de acordo com o desempenho do paciente em realizar tarefas de forma independente, com alguma ajuda ou de forma dependente. Uma pontuação geral é formada atribuindo-se pontos em cada categoria, a depender do tempo e da assistência necessária a cada paciente. A pontuação varia de 0 a 100, em intervalos de cinco pontos, e as pontuações mais elevadas indicam maior independência^(17)^.

Ainda com relação à avaliação da funcionalidade aplicou-se também o Questionário de Funcionalidade Escala de Lawton e Brody, que objetiva avaliar a autonomia do idoso na realização de atividades, necessárias para viver de maneira independente na comunidade. Avalia-se, então, a capacidade de utilizar o telefone, realizar compras, preparar refeições e trabalho doméstico, realizar viagens, administrar as finanças e usar medicamentos de forma autônoma. Cada item avaliado varia entre 1 e 3. A pontuação 3 indica que o entrevistado está em uma condição de independência; a pontuação 2 expressa uma condição de semi-dependência, o que indica que o participante necessita de ajuda parcial para as AIVD; e a pontuação 1 demonstra a existência de dependência total. Para finalizar a avaliação, somam-se os escores de todos os itens: a pontuação máxima é 21, que indica total independência por parte do indivíduo. A pontuação mínima é 7, que, por sua vez, demonstra que o avaliado é completamente dependente^(18,19)^.

Questionário de risco de declínio funcional, PRISMA 7, é composto por sete itens de autopreenchimento para identificar fragilidade. Os itens são compostos por domínios como sexo, idade, independência e funcionalidade. A resposta às perguntas é dicotômica: “sim” (1 ponto) ou “não” (0 ponto). O somatório das respostas varia entre 0 e 7, e escore ≥3 pode indicar presença de fragilidade^(20)^.

### Análise dos resultados e estatística

Os dados foram organizados em planilha do Excel e analisados por meio dos *softwares Statistical Package for the Social Science*, versão 23.0, e SAS, versão 9.4, com auxílio de profissional estatístico. Para avaliar associações entre níveis de fragilidade e variáveis qualitativas, foi aplicado o teste qui-quadrado de Pearson. Para as análises, onde foi observado resultado significativo no teste qui-quadrado e a variável qualitativa apresentava mais do que duas categorias de resposta, foram aplicados modelos de regressão Poisson modificados com variância robusta^(21)^, no intuito de identificar entre quais categorias de resposta havia diferença estatística. Nos resultados desses modelos, foram apresentadas as estimativas obtidas de Razão de Prevalência (probabilidade de vulnerabilidade), assim como os seus respectivos Intervalos de Confiança de 95%, considerando um nível de significância de 5%.

Para testar a diferença entre as médias das variáveis quantitativas, foram utilizados o teste *t* de *Student*, para amostras independentes e pareadas, teste ANOVA e teste de correlação de Pearson, e foram usados regressão linear, nos casos das variáveis com normalidade, e testes similares, em caso de estatística não paramétrica. A regressão logística foi realizada para determinar se características como capacidade funcional, estado cognitivo e presença de fragilidade são relacionadas à vulnerabilidade. Para as comparações entre os níveis de vulnerabilidade (VES-13) em relação às variáveis quantitativas, foi aplicado o teste de Mann-Whitney^(22)^. A distribuição dos dados foi avaliada por meio do teste de Shapiro-Wilk^(23)^.

## RESULTADOS

Participaram do estudo 150 idosos. A maioria (62,67%) era do sexo feminino; e 37,33% eram do sexo masculino; e 44,67% se autodeclararam brancos. A média de idade foi de 70,33 (±7,51) anos, sendo 79,34% alfabetizados. Quanto ao estado civil, 60,66% dos participantes tinham um companheiro e eram aposentados (77,33%). A renda familiar era de 2 salários mínimos para 43,33% dos participantes (Tabela 1).

**Tabela 1:** Caracterização dos participantes em relação às condições sociodemográficas, 2022 (N 150)

Variável	n	%
Sexo		
Feminino	94	62,67
Masculino	56	37,33
Cor relatada		
Branca	67	44,67
Parda	38	25,33
Preta	45	30,00
Estado civil		
Com companheiro	91	60,66
Sem companheiro	59	39,33
Nível de escolaridade		
Iletrados	31	20,67
Alfabetizados	119	79,34
Renda familiar		
1 salário mínimo (1,212,00)	36	24,00
2 salários mínimos (2,424,00)	65	43,33
≥3 salários mínimos (3,636,00)	49	32,67
Ocupação		
Aposentado	116	77,33
Cuida da casa	14	9,33
Desempregado	4	2,67
Trabalho formal	3	2,00
Trabalho informal	13	8,67

A avaliação do estado mental evidenciou que os participantes não possuíam alterações cognitivas sugestivas de déficit. Quanto à vulnerabilidade avaliada por meio do VES-13, cerca de 20,67% dos participantes estão em risco de fragilização e 38,67% já se encontram em estado de fragilidade. A aplicação da Escala de Edmonton evidencia que a maioria dos idosos possui algum grau de fragilidade e 10% dos participantes se encontram em fragilidade severa. De acordo com a escala autorreferida de fragilidade, apenas 4,67% não apresentaram nenhum grau de fragilidade (Tabela 2).

**Tabela 2:** Caracterização dos participantes com relação à preservação cognitiva, presença de fragilidade e capacidade funcional, 2022 (N=150)

Variável	n	%
Mini Exame do Estado Mental		
Iletrados (17 a 20 pontos)	32	21,33
Alfabetizados (20 a 30 pontos)	118	78
*Vulnerable Elders Survey-13*		
Não vulnerável	61	40,67
Risco de fragilização	31	20,67
Frágil	58	38,67
Escala de Fragilidade de Edmonton		
Não frágil	65	43,33
Aparentemente vulnerável	33	22,00
Fragilidade leve	25	16,67
Fragilidade moderada	12	8,00
Fragilidade severa	15	10,00
Escala autorreferida de fragilidade		
Não frágil	7	4,67
Pré-fragilidade	57	38,00
Fragilidade	86	57,33
Barthel		
Dependência em 50% de ajuda nas tarefas	3	2,00
Dependência em 25% de ajuda nas tarefas	13	8,67
Independente	134	89,33
PRISMA 7		
Não frágil	96	64,00
Frágil	54	36,00
Lawton e Brody		
Totalmente dependente	41	27,33
Dependência ligeira	48	32,00
Independente	61	40,67

Para avaliar capacidade funcional na execução das ABVD, utilizou-se a Escala de Barthel. Pôde-se observar que a maioria dos participantes foi considerada independente (89,33%), seguidos de 8,67% de idosos com dependência modificada, com a necessidade de ajuda em 25% das tarefas, e 2,00% foram considerados com dependência modificada e necessitam de ajuda em até 50% das atividades (Tabela 2).

A Escala de Lawton e Brody é utilizada para avaliar as AIVD, e, nesse domínio, 27,7% foram classificados como totalmente dependentes. A mesma proporção de idosos (27,7%) foi classificada como ligeiramente dependente, e os idosos considerados independentes foram 44,5% (Tabela 2).

Na avaliação da capacidade funcional para execução de AIVD e ABVD, ficou evidenciada a associação entre declínio funcional e risco de fragilização ou estado de fragilidade já instalado. O mesmo se observou quanto às escalas de avaliação de declínio funcional, autopercepção do estado funcional e avaliação do estado cognitivo (Tabela 3).

**Tabela 3:** Associação das condições de fragilidade e capacidade funcional, 2022 (N=150)

Variável	*Vulnerable Elders Survey-13*				Valor de *p* ^*^	Razão de Prevalência^**^	IC 95%	
	Não vulnerável		Vulnerável					
	n	%	n	%			LI	LS
Barthel					0,0030			
Dependência modificada	1	6,25	15	93,75				
Independente	60	44,78	74	55,22				
Escala de Fragilidade de Edmonton					< 0,0001			
Não frágil/aparentemente vulnerável/fragilidade leve	61	49,59	62	50,41				
Fragilidade moderada/fragilidade severa	0	0,00	27	100,00				
Lawton e Brody					< 0,0001			
Totalmente dependente	9	21,95	32	78,05		**2,65**	**1,72**	**4,07**
Dependência ligeira	9	18,75	39	81,25		**2,75**	**1,76**	**4,32**
Independente	43	70,49	18	29,51		1,00 (ref)		
PRISMA 7					< 0,0001			
Não frágil	60	62,50	36	37,50				
Frágil	1	1,85	53	98,15				
Escala autorreferida de fragilidade					< 0,0001			
Não frágil/pré-fragilidade	46	71,88	18	28,13				
Fragilidade	15	17,44	71	82,56				
Mini Exame do Estado Mental					0,0011			
Iletrados (17-20 pontos)	27	30,00	63	70,00				
Alfabetizados (20-30 pontos)	34	56,67	26	43,33				

* Valor de p obtido por meio do teste qui-quadrado de Pearson; IC - Intervalo de Confiança; LI - limite inferior; LS - limite superior;

** Resultados obtidos por meio de modelo de regressão Poisson modificado. Foi estimada a probabilidade de apresentar o resultado “risco de fragilização/frágil”.

Observou-se forte associação entre dependência em ABVD e AIVD e risco de fragilização ou estado de fragilidade instalado. O estudo evidenciou que idosos identificados com dependência ligeira têm 2,75 vezes maior probabilidade de risco de fragilização, ao passo que idosos totalmente dependentes têm 2,65 vezes maior probabilidade de fragilização (Tabela 3).

## DISCUSSÃO

O presente estudo analisou a associação entre vulnerabilidade e níveis de fragilidade e funcionalidade de idosos assistidos pela APS. Por meio dele, foi possível inferir que existe forte associação entre declínio funcional e cognitivo e risco de vulnerabilidade e fragilidade. Além disso, observou-se a presença de dependência desses idosos para ABVD e AIVD e risco de fragilização desses idosos.

A fragilidade severa identificada neste estudo afeta 10% da população participante, e 38,67% se encontravam em estado de vulnerabilidade. A presença de fragilidade está associada a alto risco para a ocorrência de problemas relacionados à saúde, uma vez que a fragilidade está casualmente relacionada a múltiplas condições crônicas e suas consequências diversas, como diminuição do equilíbrio e marcha, fraqueza muscular e fadiga, perda de peso, declínio funcional e cognitivo, deixando a pessoa idosa em estado de elevada vulnerabilidade e em risco para o desenvolvimento da fragilidade^(24,25)^.

A síndrome de fragilidade afeta, primeiramente, a saúde do indivíduo, sua capacidade funcional e cognitiva, fator este que dificulta a execução de atividades de gerenciamento doméstico, isto é, as AIVD. AIVD exigem maior preservação física e cognitiva para sua execução; por isso, seu declínio antecede o declínio da funcionalidade para execução das atividades de autocuidado, em outras palavras, as ABVD. Em consequência da evolução da síndrome de fragilidade e do declínio funcional, associado a outras comorbidades, o idoso torna-se gradativamente dependente, perde autonomia e pode ficar mais susceptível ao agravo de doenças, situações de risco para vulnerabilidade e fragilização^(26,27)^.

Estudos pregressos evidenciaram que idosos com capacidade funcional reduzida apresentam maior dependência para execução das AIVD e risco de fragilização. É possível observar maior prevalência de dependência nas atividades de gestão doméstica. Neste estudo, 27,33% dos idosos estão dependentes em AIVD. A dependência nessas atividades pode ser determinante para o avanço do declínio funcional e cognitivo. Consequentemente, idosos com incapacidades na execução das AIVD estão sujeitos a inúmeros fatores que os expõem a riscos de fragilização e agravos de saúde como a síndrome de fragilidade^(27,28)^.

O estudo evidenciou, ainda, que 10,67% dos participantes são dependentes em atividades de autocuidado. Declínio funcional é um fator determinante para dependência, necessidade de cuidados e desenvolvimento da síndrome de fragilidade. Estudos pregressos corroboram e evidenciam que declínio funcional está associado à síndrome da fragilidade. De acordo com estudo realizado no município de Ribeirão Preto, SP, foi possível observar que, das 148 pessoas idosas que participaram do estudo, 81,9% apresentaram fragilidade e dependência parcial nas AIVD e, consequentemente, em ABVD^(28)^.

Em estudo pregresso realizado por Neri^(29)^ no município de Campinas, SP, foram encontrados resultados que corroboram os achados do presente estudo, em que é evidenciada a forte associação entre declínio cognitivo e risco de fragilização ou fragilidade já instalada. A pessoa idosa é mais propensa à exposição a fatores de vulnerabilidade e acometimento por doenças crônicas e agravos como a síndrome de fragilidade, que por sua vez pode ter desfechos indesejáveis, como adoecimento, declínio funcional e cognitivo, institucionalização, hospitalização, e morte^(29,30)^.

A vulnerabilidade da pessoa idosa é multifatorial e, por isso, recomenda-se a vigilância das condições que propiciam maior risco de desenvolvimento da fragilidade^(31)^. Conhecer as condições associadas à vulnerabilidade e fragilização da pessoa idosa na comunidade pode proporcionar melhor entendimento sobre as ações e estratégias necessárias para prevenção do declínio cognitivo e funcional^(32)^. Além disso, este estudo pode contribuir para futuros estudos longitudinais para avaliar as comparações realizadas no presente estudo e intervenções para melhoria da fragilidade e vulnerabilidade na população idosa.

Diante do exposto, é essencial que haja investimento governamental e gestão adequados para atender às necessidades dessa população crescente, buscando adequar e garantir acesso aos serviços de saúde e ações de promoção de saúde e prevenção de agravos. Urge providenciar, simultaneamente, melhorias na qualificação dos profissionais, principalmente quanto à vigilância e estratificação de risco da pessoa idosa para identificação de fatores que possam desencadear a síndrome de fragilidade. Viabilizar ações que reduzam a vulnerabilização e permitam envelhecimento saudável dessa parcela crescente da população brasileira exige a tomada de decisão na direção da garantia de acesso e combate às iniquidades em saúde.

### Limitações do estudo

Contudo, é apropriado considerar algumas limitações do estudo, entre as quais destaca-se que, apesar de ser estudo multicêntrico, os resultados aqui apresentados são de apenas um dos municípios participantes, uma vez que a coleta de dados continua a ser realizada em outros municípios brasileiros, assim como em outros países. Ressalta-se, também, a seleção dos participantes por conveniência entre aqueles que frequentavam as unidades de APS, tendo como viés os idosos do sexo feminino, uma vez que são os usuários mais frequentes nesses espaços. Finalmente, as informações passadas pelos próprios idosos podem representar vieses, principalmente aquelas relativas à autopercepção, uma vez que são questões subjetivas e de difícil verificação.

### Contribuições para as áreas da saúde, enfermagem, ou políticas públicas

O conhecimento sobre a situação de risco para vulnerabilidade, fragilidade e declínio funcional dos idosos neste município contribui para conhecimento dos aspectos associados à vulnerabilidade e fragilidade da pessoa idosa. Por meio desse conhecimento, é possível desenvolver ações para promoção do envelhecimento ativo e saudável, e contribuir para conhecimento dos profissionais de saúde da APS.

## CONCLUSÕES

Este estudo possibilitou analisar a relação e associação das condições de vulnerabilidade e fragilidade com o declínio funcional da pessoa idosa assistida pela APS em um município do Centro-Oeste de Minas Gerais. Foi evidenciada associação da presença de vulnerabilidade em idosos com maior nível de dependência nas ABVD e AIVD e a presença ou risco de fragilidade.

É importante investimento para qualificação dos profissionais de saúde, para que sejam capazes de identificar previamente as condições sociais e de saúde que resultam em declínio funcional e cognitivo e, por fim, na síndrome de fragilidade e para que possam traçar estratégias de prevenção da fragilidade na pessoa idosa.

## Data Availability

Os dados da pesquisa estão disponíveis no corpo do artigo.
